# Supratrochlear Rim Resection: Autologous Matrix-Induced Chondrogenesis–Based Arthroscopic Technique for Patients With Isolated Patellar Chondromalacia

**DOI:** 10.1016/j.eats.2025.103571

**Published:** 2025-05-10

**Authors:** Tomasz Piontek, Bartosz Bąbik, Jakub Bąbik, Artur Banach

**Affiliations:** a Rehasport Clinic/Department of Spine Disorders and Pediatric Orthopedics, University of Medical Sciences, Poznan, Poland; b Department of Orthopaedics, Pediatric Orthopaedics and Traumatology, Medical Centre of Postgraduate Education, Otwock, Poland; c Faculty of Architecture, Warsaw University of Technology, Warsaw, Poland; d MicroBioRobotic Systems Laboratory, Institute of Mechanical Engineering, École Polytechnique Fédérale de Lausanne, Lausanne, Switzerland

## Abstract

Isolated patellar chondromalacia in patients with patella alta and no history of patellar subluxations or dislocations is associated with a supratrochlear rim at the entrance to the femoral trochlea. This technical note outlines an arthroscopic technique called supratrochlear rim resection, which proposes a line of treatment that addresses not only symptoms (patellar cartilage defect) but also a suspected biomechanical reason (supratrochlear rim). The proposed technique combines resection of the supratrochlear rim with a modification of autologous matrix-induced chondrogenesis cartilage reconstruction on the femoral side at the location of the resected rim, as well as on the patellar defect.

Anterior knee pain, commonly referred to as “patellofemoral pain” (PFP), accounts for approximately 50% of knee pain cases in adolescents, with around 30% of this population affected by knee pain.^1^, ^2^, ^3^ Ineffective management of PFP can lead to isolated patellar chondromalacia (PC), a degenerative condition of the patellar cartilage (Fig 1).^4^^,^^5^ The etiology of isolated PC is well established in individuals with a history of patellar subluxation or dislocation, particularly in cases of severe trochlear dysplasia.^6^ However, the pathogenesis of isolated PC in patients without a history of patellar subluxation or dislocation remains unclear.^7^ It may result from structural and functional abnormalities within the patellofemoral joint, particularly abnormal patellar tracking within the trochlear groove, which could irritate highly innervated structures such as the subchondral bone and lateral retinaculum.^8^^,^^9^

**Fig 1:**
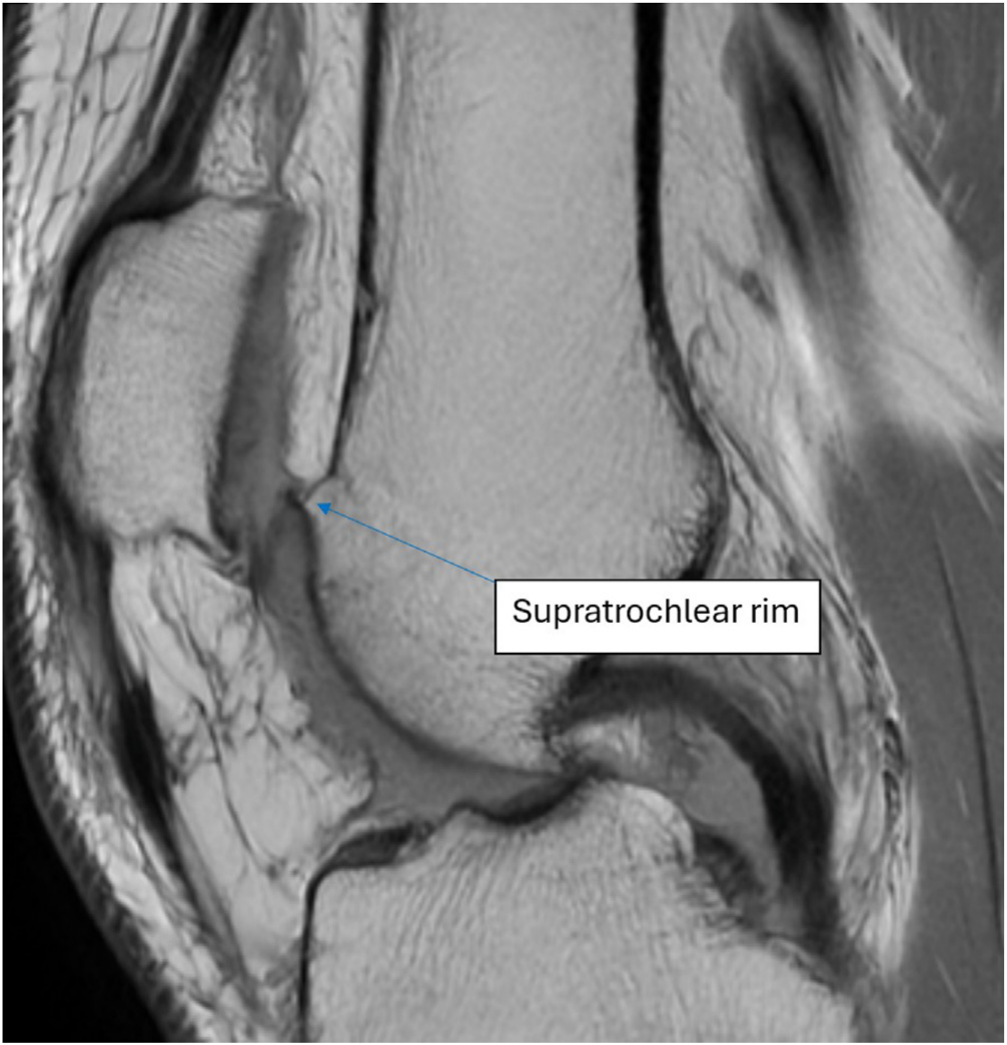
Supratrochlear rim on magnetic resonance imaging in sagittal view. The arrow is pointing to the bony supratrochlear rim. The presented cross section is the slice in which the anterior contour of the medial femoral condyle becomes visible, along with the rim. Magnetic resonance imaging diagnostics of the knee joint are required to confirm the presence of the rim before planning the surgical procedure. This imaging step also assesses the state of the patellar cartilage.

Outerbridge^10^ suggested that abnormal patellar tracking and alignment in patients without a history of subluxation or dislocation could be due to an exaggerated curvature of the anterior femur at the proximal entrance to the trochlea, an abnormality referred to as a “supratrochlear rim.” Subsequent studies have confirmed this association and introduced an open surgical approach to address the problem by removing excess bone beneath the femoral cartilage, thereby eliminating the rim, restoring a smooth femoral condyle contour, and yielding satisfactory initial clinical outcomes.^11^, ^12^, ^13^, ^14^

This report presents an arthroscopic technique called supratrochlear rim resection for patients with isolated PC and no history of patellar subluxations or dislocations. The presented technique smoothens the femoral contour and reconstructs the patellar cartilage defect to improve patellar tracking and to reduce PFP.

## Surgical Technique

Supratrochlear rim resection is described and illustrated in this section and in Video 1.

### Indications for Rim Resection

**Fig 2:**
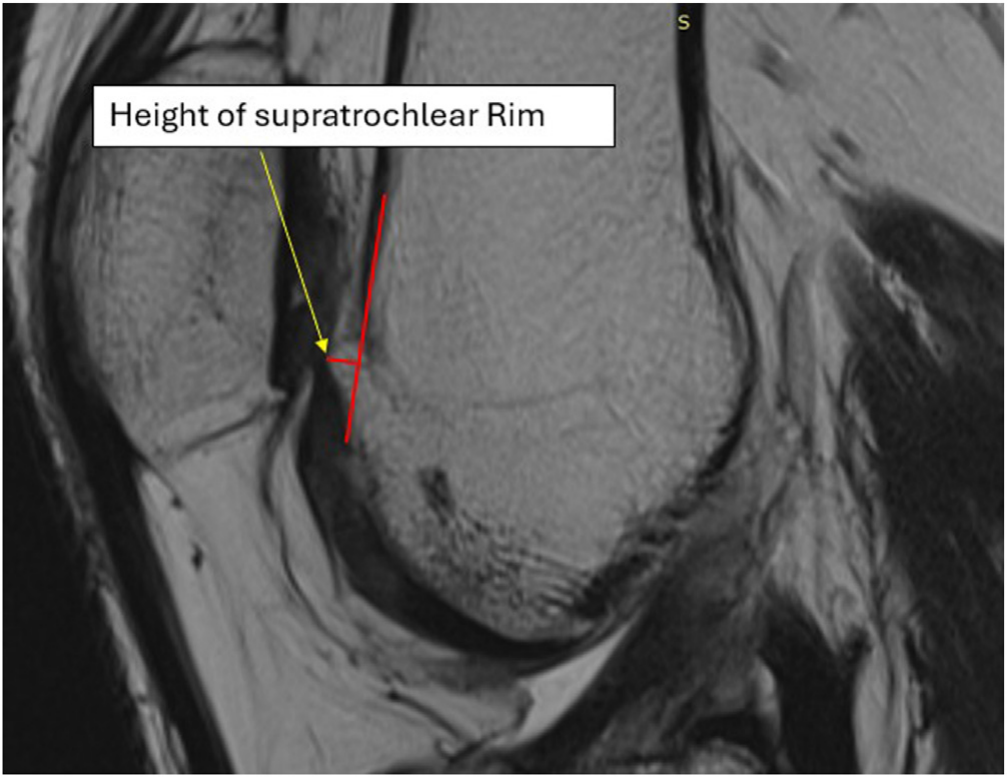
Magnetic resonance imaging sagittal slice showing measurement of height of supratrochlear rim. The measurement is taken on magnetic resonance imaging in the sagittal view, in which the deepest point of the trochlear groove is visible. The rim height (short red line) is measured from the extension of the frontal femoral cortex (long red line) to the most prominent anterior point of the supratrochlear rim.

The indications for supratrochlear rim resection include a supratrochlear rim identified on magnetic resonance imaging in the sagittal view (Fig 1); no history of patellofemoral joint subluxations or dislocations; patella alta (patellotrochlear index < 0.125-0.28)^15^^,^^16^; PFP during the first 30° of knee flexion from full extension; and a supratrochlear rim height of up to 3 mm measured in the sagittal plane (Fig 2) (if the height exceeds 3 mm, the patient should be qualified for a different procedure). The exclusion criteria include active infection of the knee joint or the operated limb and a history of patellar subluxations or dislocations.

### Supratrochlear Rim Resection

**Fig 3:**
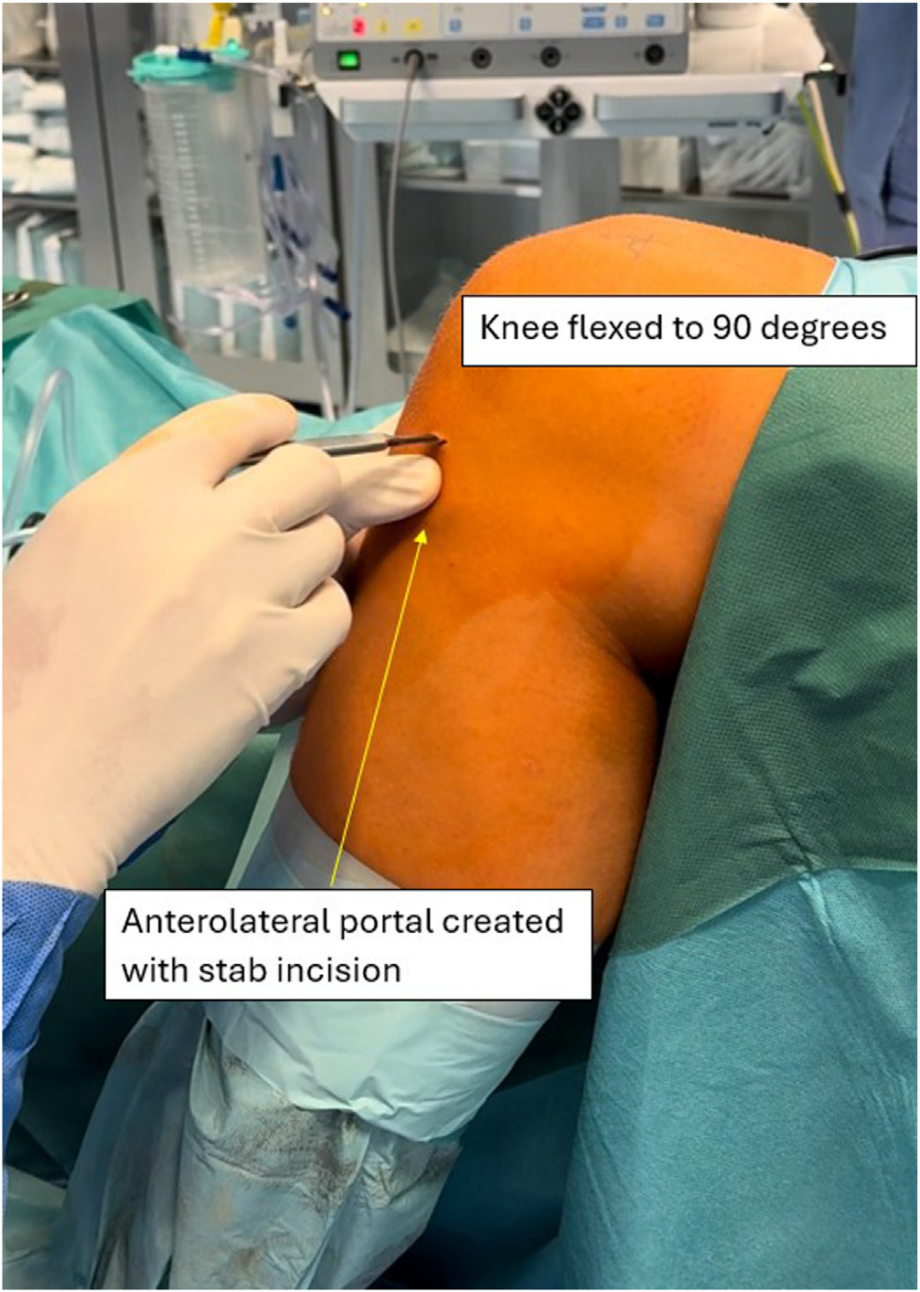
The anterolateral portal is created with a stab incision using a scalpel in the left knee joint. The leg is positioned at 90° of flexion. The patient is positioned supine, with an Esmarch band applied. The access point is made at the soft spot lateral to the patellar ligament. The arrow indicates the created anterolateral portal. The surgeon is seated, with the patient’s foot resting on the surgeon’s lap.

**Fig 4:**
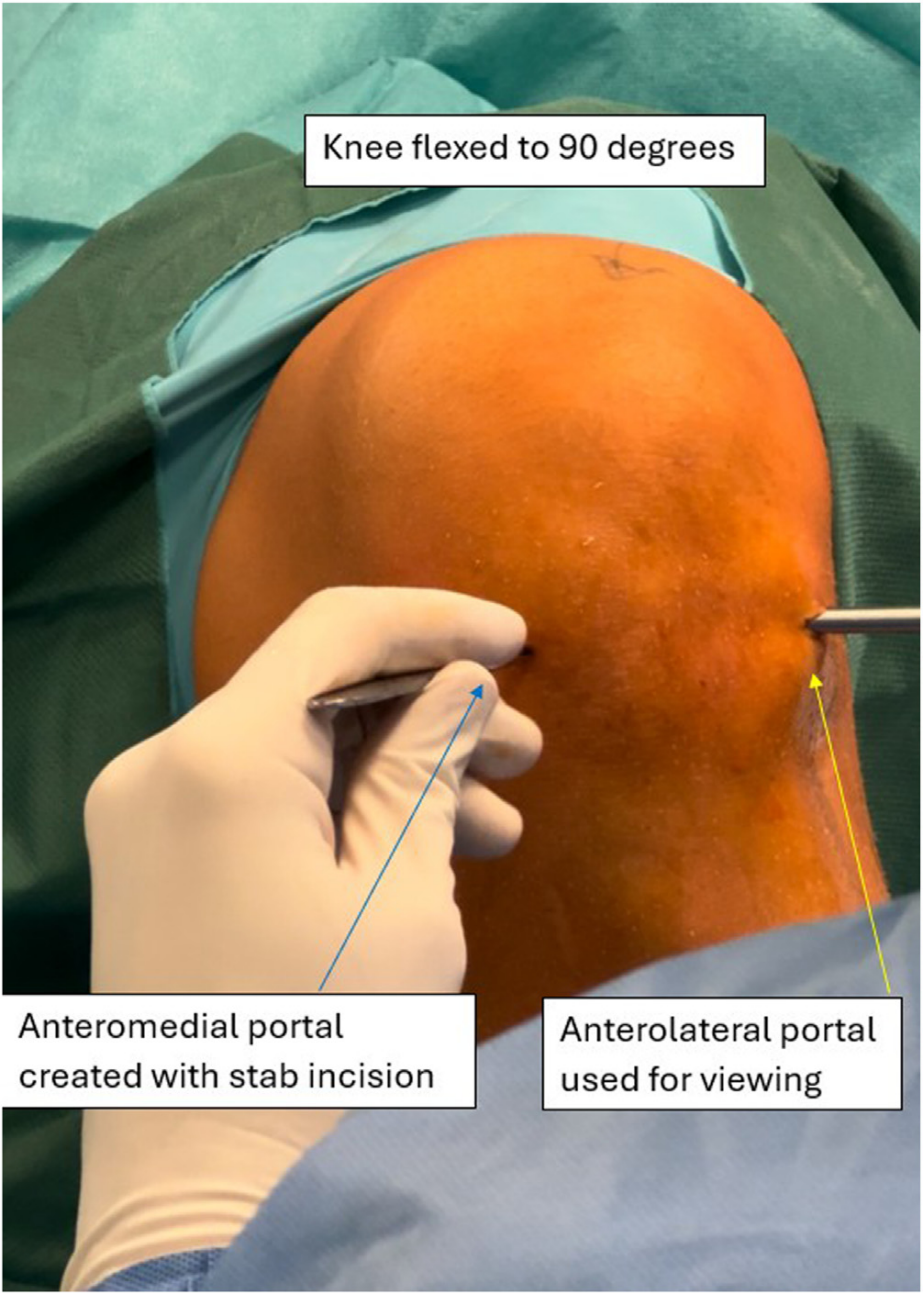
An anteromedial portal is created with a stab incision in the left knee. The position is confirmed visually via the anteromedial portal using a needle. The patient is positioned supine, with an Esmarch band applied. During portal creation, the knee is flexed to 90°. The access point is made medial to the patellar ligament. The yellow arrow points to the arthroscopic camera inserted through the previously created anterolateral portal. The blue arrow points to the surgical knife used to create the anteromedial portal. The surgeon is seated, with the patient’s foot resting on the surgeon’s lap.

**Fig 5:**
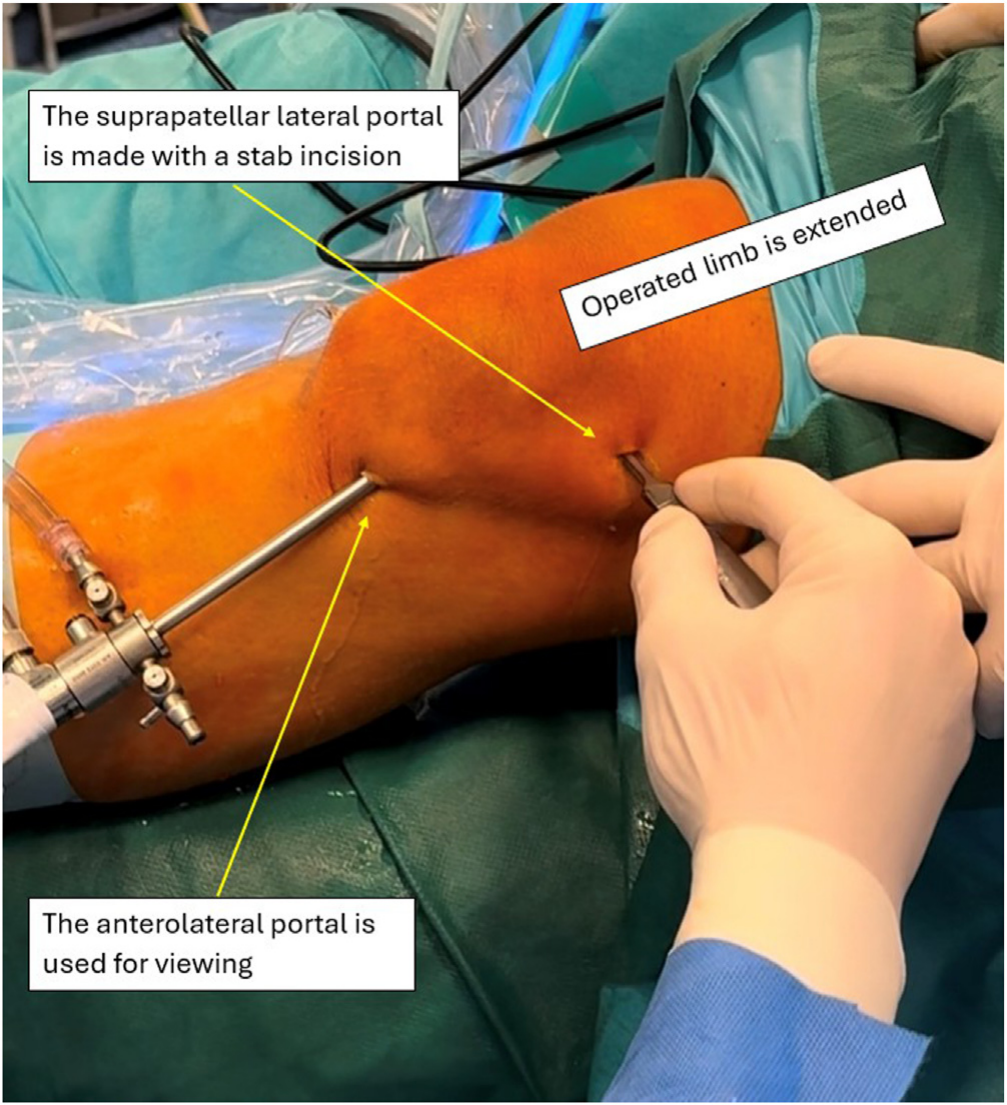
Left operated limb lying on operating table in full extension. After the anterolateral and anteromedial portals are created, with the use of a probing needle and viewing through the previously created anterolateral portal, the lateral suprapatellar portal is created with a stab incision under arthroscopic visualization.

**Fig 6:**
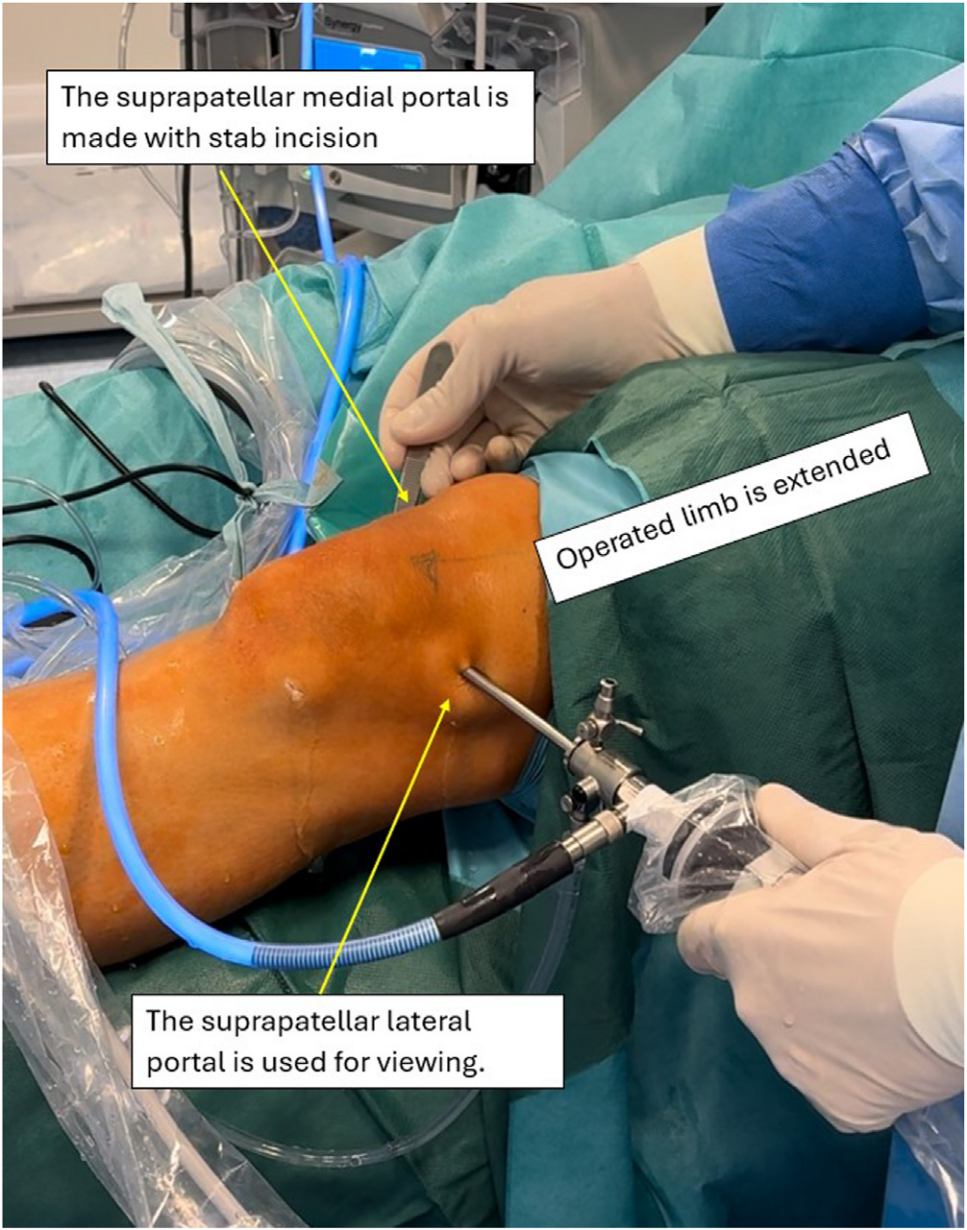
Left operated limb lying on operating table in full extension. The medial suprapatellar portal is created with a stab incision under direct arthroscopic visualization via the lateral suprapatellar portal. These additional portals will later serve as viewing (lateral) and working (medial) portals for the rim resection. The lower arrow indicates the arthroscopic camera inserted through the lateral suprapatellar portal. The upper arrow points to the medial suprapatellar portal being created.

The patient is positioned supine, with an Esmarch tourniquet applied proximally to the thigh and inflated to 300 mm Hg of pressure. The procedure is performed under spinal anesthesia. A disposable surgical drape is applied, and the operative field is prepared aseptically. A standard anterolateral (AL) portal is created (Fig 3), followed by an anteromedial (AM) portal (Fig 4), both made with a stab incision using a scalpel. The AM portal is made under visualization with an arthroscope introduced via the AL portal. All concomitant pathologies in the knee joint are addressed and treated first. Then, with the knee held in extension, a probing needle is used to establish the correct placement for the creation of a lateral suprapatellar portal under arthroscopic visualization via the AL portal. The lateral suprapatellar portal is subsequently made with a stab incision (Fig 5). Next, the medial suprapatellar portal is created in a similar way. Visualization for correct placement of the medial suprapatellar portal may be from the AL portal or the newly created lateral suprapatellar portal. These additional portals will later serve as viewing and working portals (Fig 6).

The patellar defect is inspected to determine its extent. By use of an arthroscopic hook, the defect’s margins are delineated. Resection is performed with front and sideward Chondrectom instruments (Chondrectom Extended Set; Biovico, Gdynia, Poland) as needed to the boundaries of healthy cartilage, followed by smoothing with an arthroscopic shaver. The resection proceeds to the subchondral bone. The patellar defect is managed through the AL portal as a viewing portal and the AM portal as a working portal. Depending on the location of the defect, the remaining portals can also be used.

**Fig 7:**
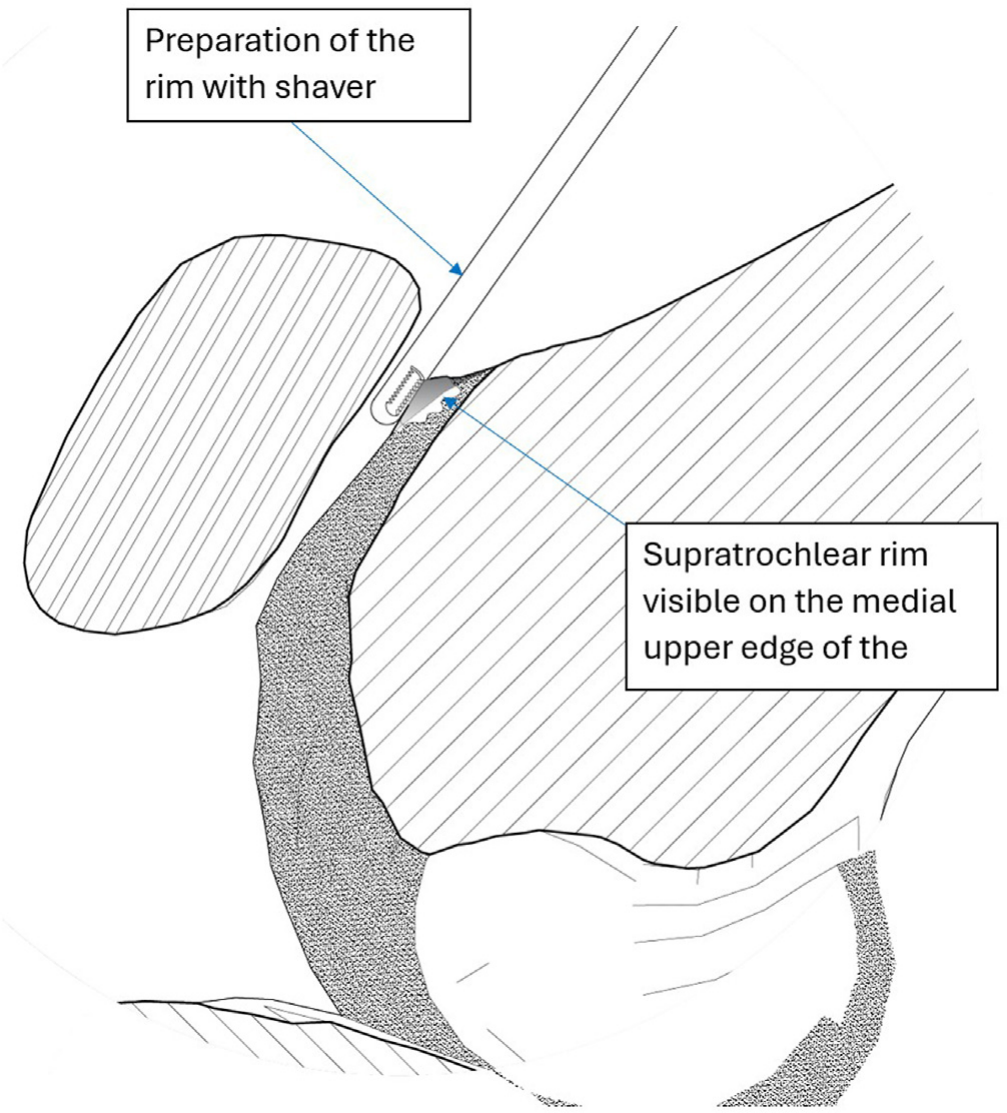
Preparation of supratrochlear rim. The cartilage overlying the supratrochlear rim is resected with a shaver to the subchondral bone, exposing the bony rim on the medial side of the trochlear groove. The medial suprapatellar portal serves as the working portal, while the lateral suprapatellar portal functions as the viewing portal. During rim preparation, the operated limb rests on the foot in full extension.

**Fig 8:**
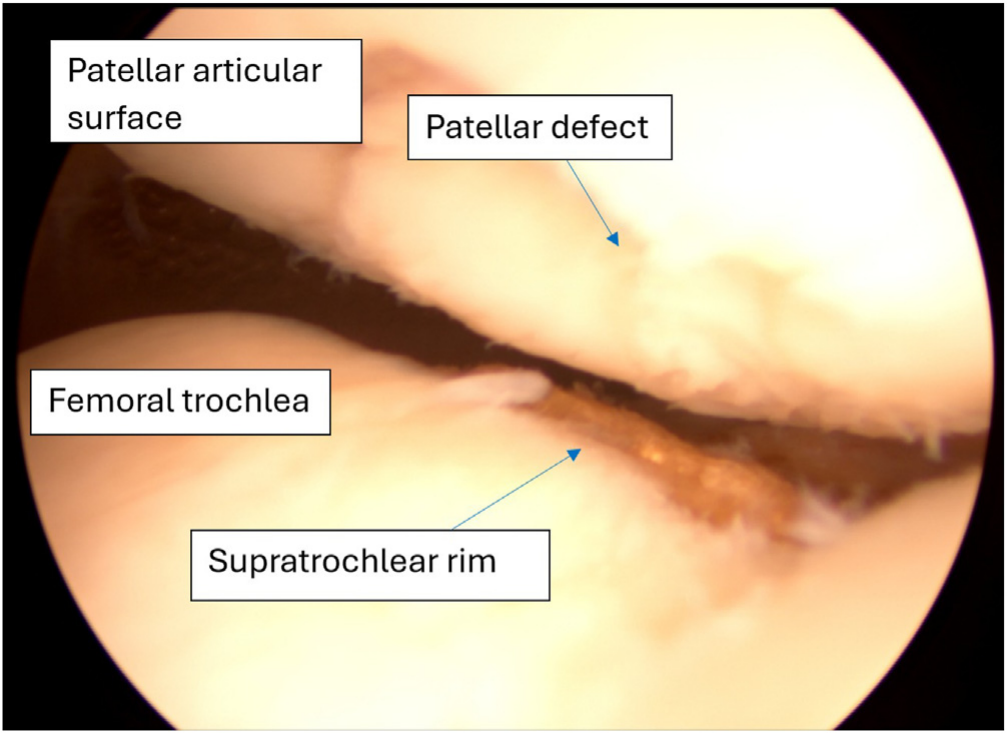
Arthroscopic image in left knee visualizing correlation between supratrochlear rim and patellar chondral defect during dynamic test. The knee is flexed to 30°, and the patella is pressed against the femur. In this case, the image is captured from the arthroscopic camera inserted through the anterolateral portal.

**Fig 9:**
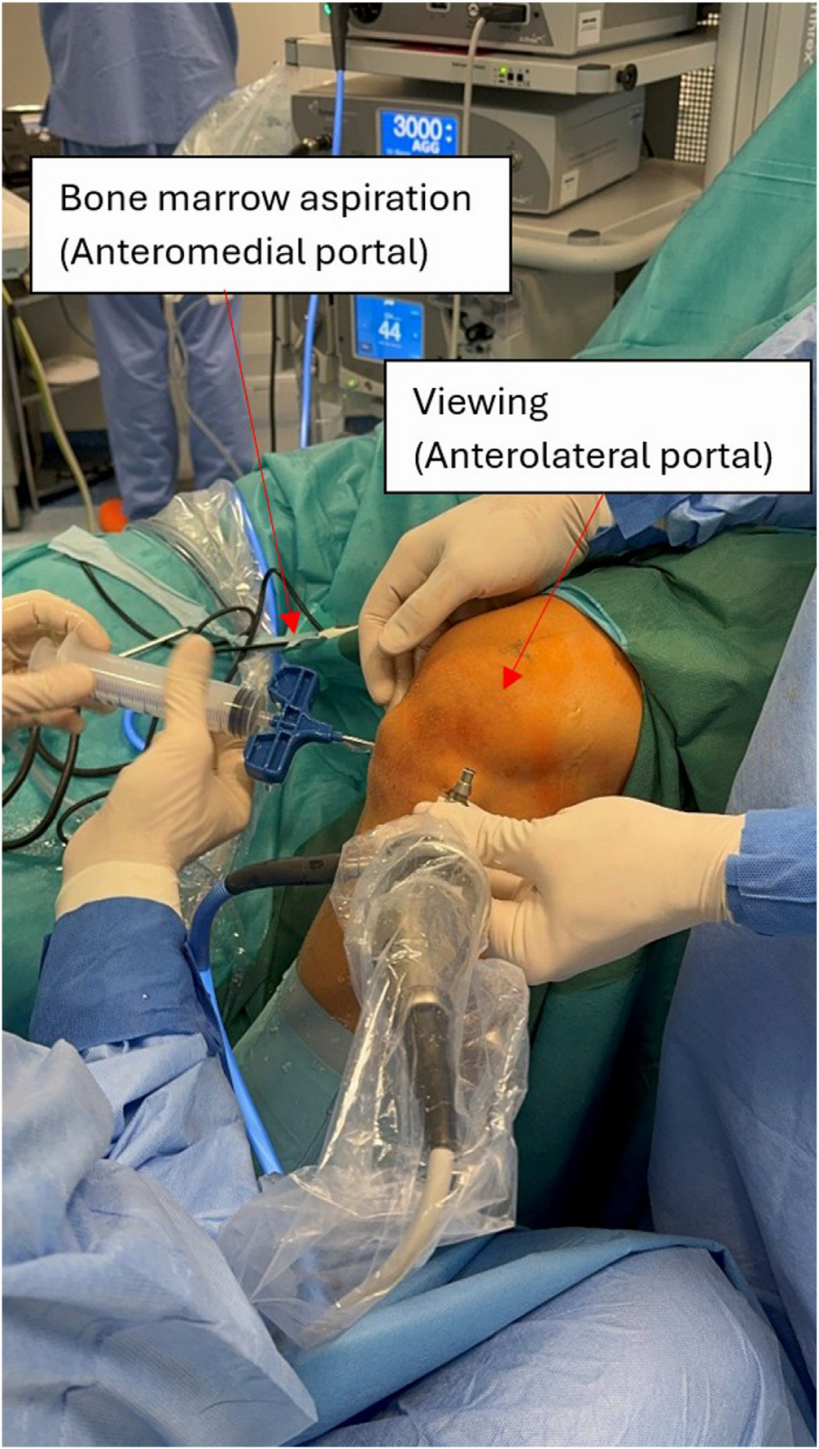
Bone marrow is aspirated from the intercondylar notch. The needle is introduced via the anteromedial portal under visualization from the anterolateral portal. The leg is flexed to approximately 90°. The arrows indicate the bone marrow aspiration cannula inserted through the anteromedial portal and the arthroscopic camera introduced through the anterolateral portal.

**Fig 10:**
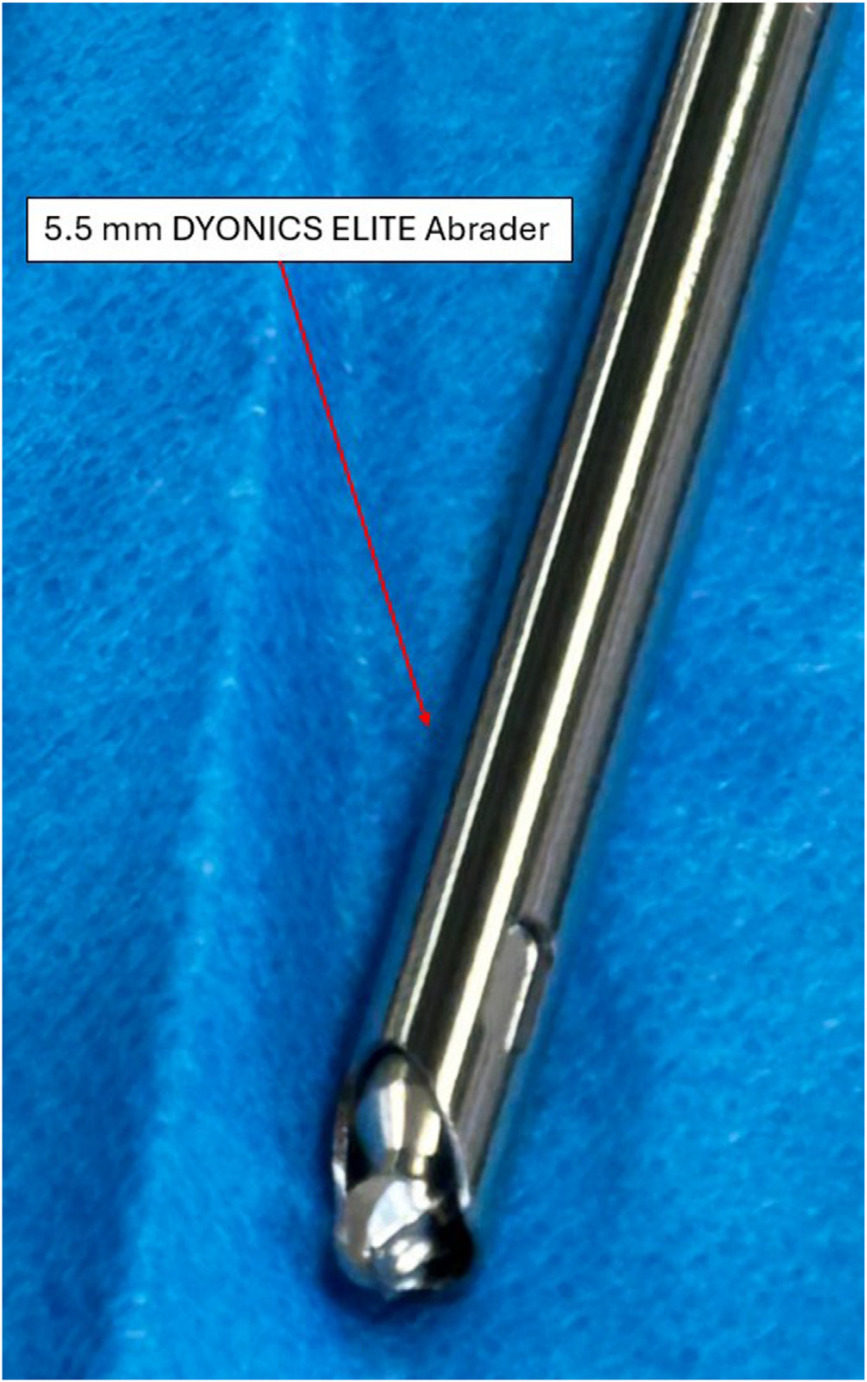
Straight burr (5.5-mm Dyonics Elite Abrader) used for supratrochlear rim resection. The burr is essential for performing resection of the bony supratrochlear rim. It is introduced through the medial suprapatellar portal.

**Fig 11:**
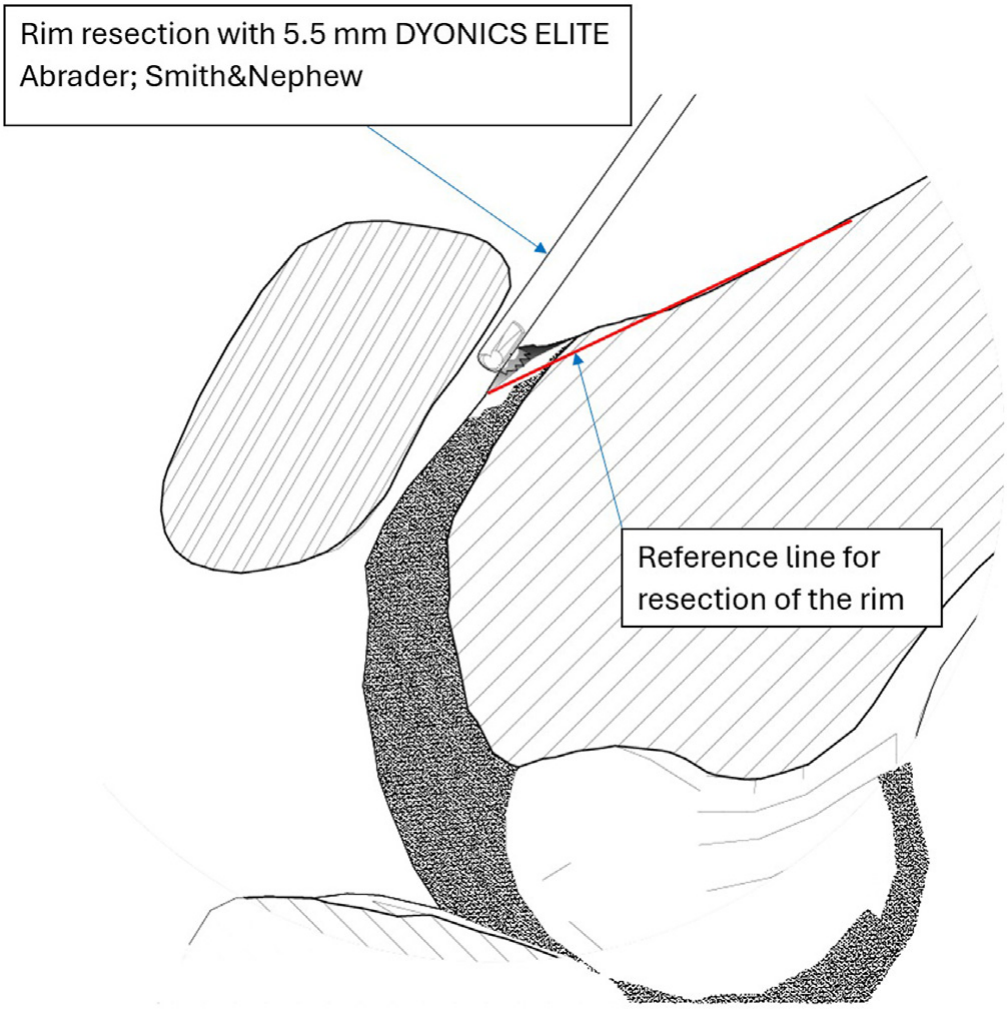
Sagittal section of knee joint. After preparation, the supratrochlear rim is resected using a straight burr (5.5-mm Dyonics Elite Abrader). The red line along the extension of the anterior femoral cortex defines the resection boundary. During rim resection, the patient’s limb remains in a fully extended position on the operating table. The surgeon faces the patient’s feet. The lateral suprapatellar portal serves as the viewing portal, while the burr (Dyonics Elite Abrader) is introduced through the medial suprapatellar portal.

The supratrochlear rim is identified and prepared. Both preparation of the supratrochlear rim and then its resection are performed with the medial suprapatellar portal as a working portal and the lateral suprapatellar portal used for viewing. Soft tissues overlying the anterior femoral cortex are initially resected with an electrocautery device, extending to the transition zone between the cortex and the trochlear groove to ensure complete visualization. Next, the cartilage overlying the supratrochlear rim is resected with a shaver and electrocautery device to the subchondral bone, exposing the bony rim on the medial side of the trochlear groove (Fig 7). Passive dynamic knee flexion is used to evaluate the correlation between the patellar defect and the supratrochlear rim (Fig 8). The rim is then resected using a straight burr (5.5-mm Dyonics Elite Abrader; Smith & Nephew, Watford, England) (Fig 9). The outline of the anterior femoral cortex is used as a reference line (Fig 10). Rough edges are smoothed with a regular shaver. For bone marrow aspiration, a needle is introduced through the AM portal to the intercondylar notch, and bone marrow aspirate is obtained from the lateral condyle with visualization through the AM portal (Fig 11).

**Fig 12:**
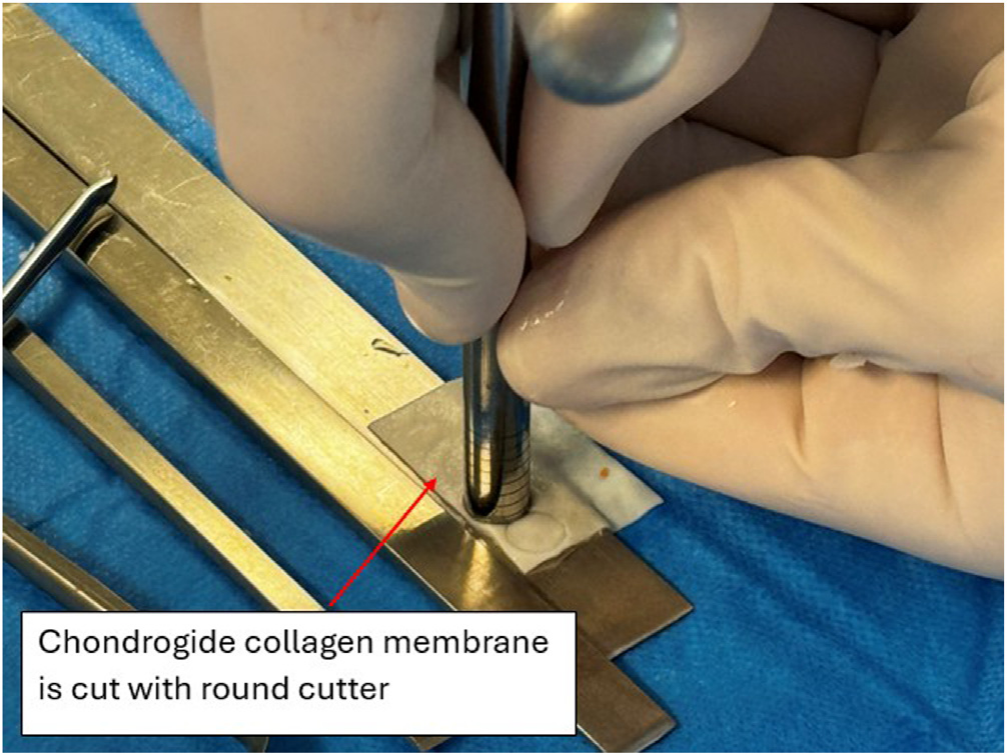
Preparation of collagen membrane (arrow). Round and semi-round pieces of collagen membrane (Chondro-Gide) are cut with the round cutter (OBI reconstruction kit).

**Fig 13:**
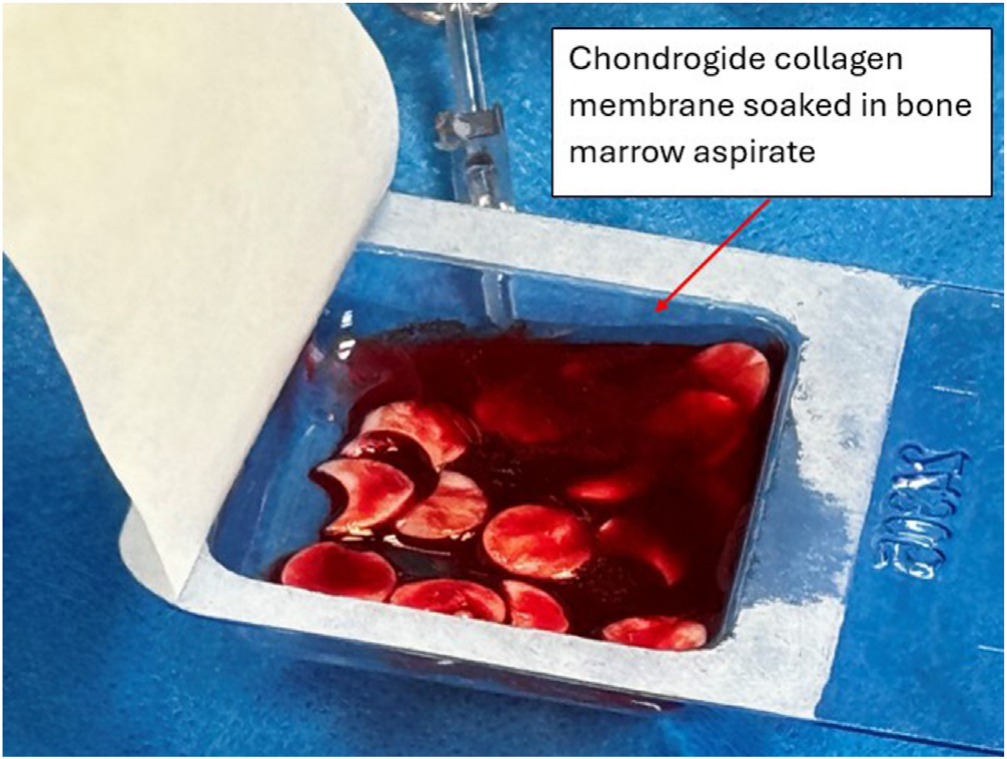
All collagen membrane pieces are soaked in previously acquired blood marrow aspirate. The pieces are prepared for implantation using dry arthroscopy.

**Fig 14:**
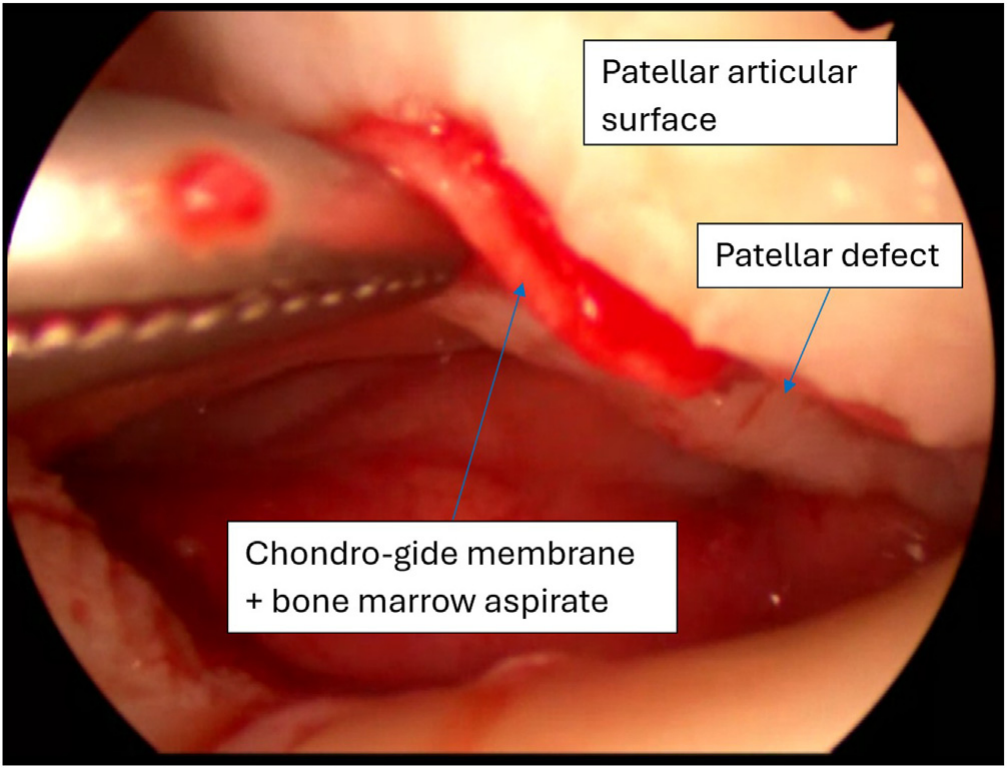
Arthroscopic view of knee joint. The soaked collagen membrane pieces are introduced with a pean clamp via the anteromedial portal into the patellar defect under dry arthroscopy. The anterolateral portal is used as the viewing portal. The leg is lying on the surgical table in full extension.

**Fig 15:**
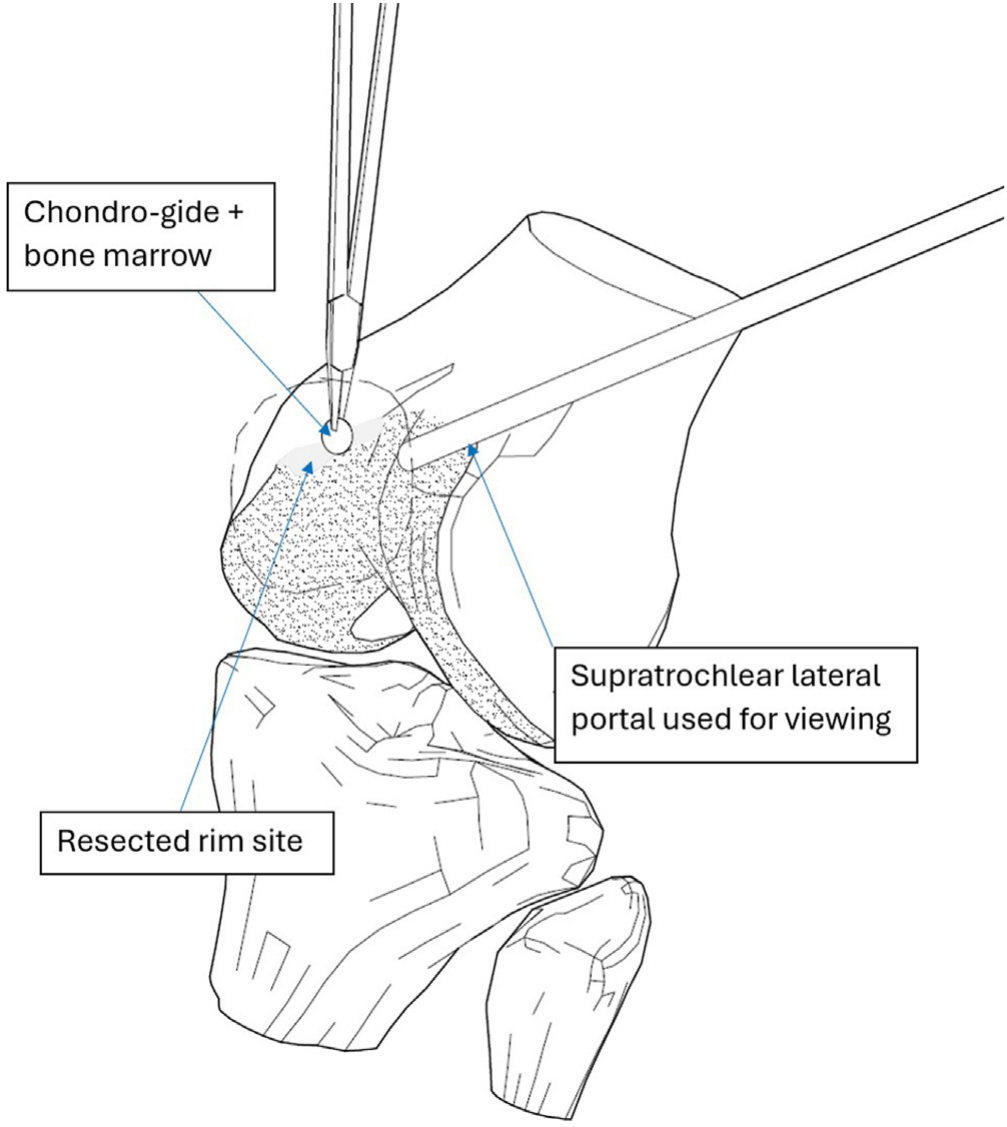
Resected rim site. The soaked collagen membrane pieces are introduced with a pean clamp via the medial suprapatellar portal onto the femoral surface in place of the resected supratrochlear rim under dry arthroscopy. The lateral suprapatellar portal is used for viewing. The leg is lying on the surgical table in full extension.

**Fig 16:**
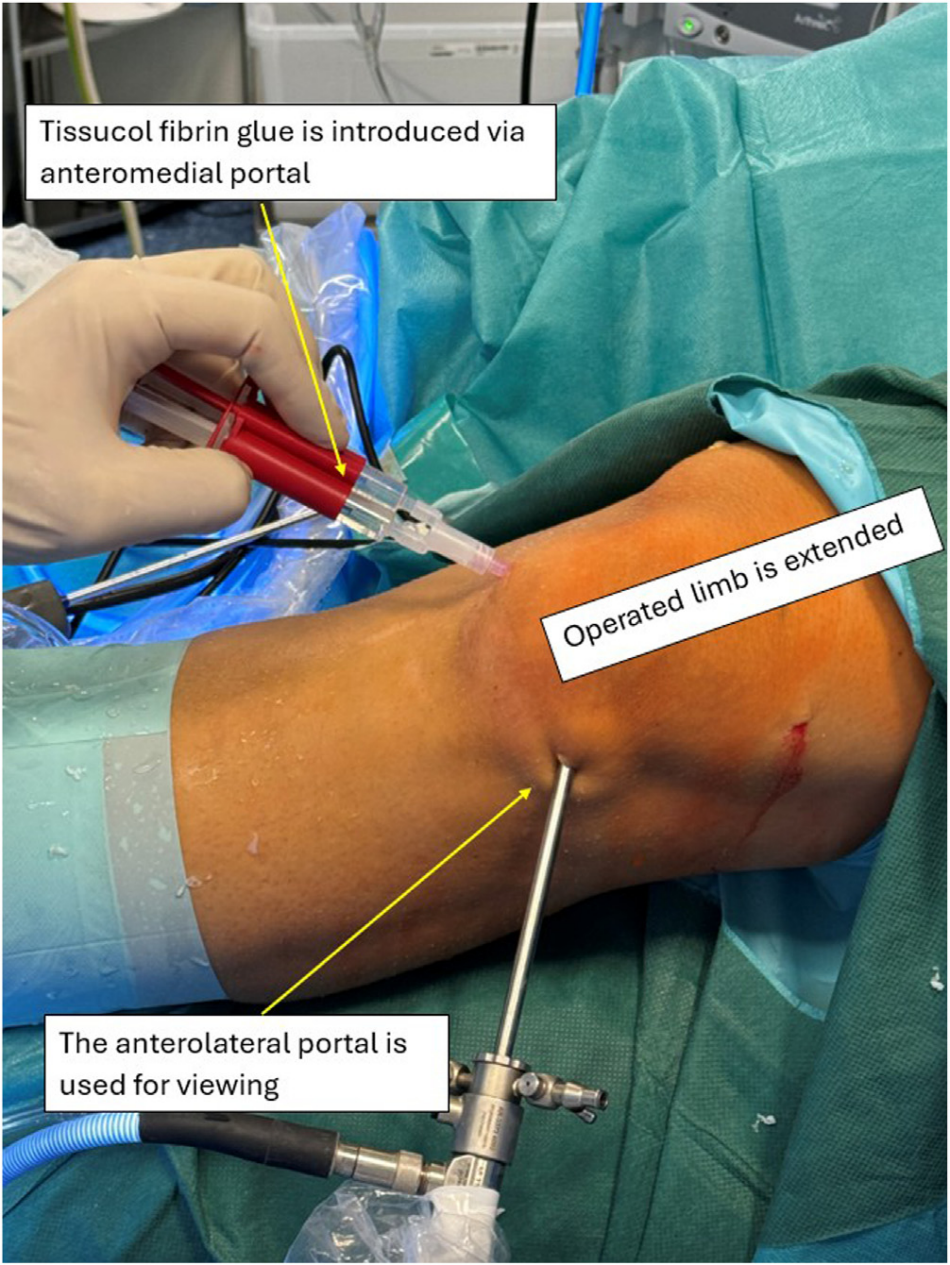
Fibrin glue implementation. After implantation of all the collagen membrane pieces on both surfaces, fibrin glue (Tissucol) is used for final stabilization of the membrane also on both surfaces. The leg is lying on the operating table in full extension. The glue is introduced via the anteromedial portal; the anterolateral portal is used for viewing.

Meanwhile, a 20 × 30-mm collagen membrane (Chondro-Gide; Geistlich Pharma, Wolhusen, Switzerland) is cut with a round cutter (OBI reconstruction kit; Smith & Nephew). Round and semi-round pieces (5 × 5 mm) of collagen matrix are cut from the matrix (Fig 12) and soaked in the previously obtained bone marrow aspirate (Fig 13). The next part of the procedure is performed under dry arthroscopy. An arthroscopic autologous matrix-induced chondrogenesis (AMIC) technique is used for cartilage reconstruction in this procedure.^17^ Instead of microfractures, described in the original method, bone marrow aspirate is used (modification of the AMIC technique called AMIC+).^18^ By use of a pean clamp, the round Chondro-Gide pieces are placed on the previously prepared site of the chondral patellar defect (Fig 14) and on the femoral surface in place of the resected supratrochlear rim (Fig 15). Then, both reconstructed surfaces are stabilized with fibrin glue (Tissucol; Baxter, Warsaw, Poland) (Fig 16). The fibrin glue is introduced through the AM or medial suprapatellar portal, with visualization through the AL or supratrochlear lateral portal. It is important to prevent glue adherence to surrounding tissues; if adhesion occurs, it should be excised using arthroscopic scissors to minimize potential complications. Once the glue is dry, 10 cycles of passive knee flexion and extension are performed to assess the stability of the reconstructed cartilage surfaces. Final assessment is conducted using dry arthroscopy to evaluate the repair’s integrity and alignment. Once stability of the reconstructed surfaces is confirmed, the procedure is concluded. The surgical wounds are closed without drainage, and a layered dressing is applied. Pearls and pitfalls of the presented technique are listed in Table 1.

**Table 1:** Pearls and Pitfalls of Presented Technique

Pearls
During rim preparation, the camera and instruments should be positioned in superior portals. The surgeon should be facing the foot and adjust his or her position as needed.
It is essential to remove the synovium from the suprapatellar recess to ensure that it does not obstruct visualization during dry arthroscopy.
During switching of surgical portals, guides and cannulas should be used to allow smooth insertion of the instruments into the knee joint.
After fibrin glue is applied to the reconstructed cartilage surface, it is essential to verify that the adhesive does not bond the implanted collagen membrane to the synovium. If adhesion occurs, scissors should carefully separate the glue from the synovium. This step needs to be performed under dry arthroscopy.
Pitfalls
Improperly positioned surgical portals may hinder free instrument manipulation within the knee joint.
Collagen membrane that extends beyond the lesion onto healthy tissue leads to improper fixation. During joint movement, the membrane may shift, compromising the reconstruction.
The joint should not be refilled with saline solution after membrane implantation and adhesive application.

### Rehabilitation Protocol

After the surgical procedure, the patient starts knee flexion using a continuous passive movement device on the second day and continues for approximately 6 hours a day until 2 weeks postoperatively. The maximum flexion angle on the continuous passive movement device should be as high as possible without the patient experiencing any pain. Sutures are removed 2 weeks after surgery. The patient walks with elbow crutches for up to 6 weeks after surgery. The patient may return to running after achieving full range of motion, no pain or leakage, and everyday functional parameters symmetrical with the nonoperated limb, such as strength and endurance.

## Discussion

Treatment of patellofemoral joint disorders should be planned and individualized for each patient. Dejour et al.^19^ emphasized the importance of diagnosing and treating the causes of patellofemoral joint instability.^20^, ^21^, ^22^ In patients without patellar instability, addressing PC alone, without correcting other structural abnormalities, may not yield positive outcomes.^14^^,^^23^, ^24^, ^25^, ^26^, ^27^ Our surgical technique is intended for patients without clinical signs of patellofemoral instability. In the presented procedure, we use the arthroscopic AMIC+ technique, which has yielded good clinical results in treating chondral defects for nearly 2 decades.^17^^,^^28^ Therefore, it is expected that AMIC+ of the reconstructed femoral surface after rim resection will also yield positive clinical outcomes.^18^^,^^29^

The proposed technique is a complex arthroscopic procedure that requires a highly skilled operator and a set of dedicated tools for both rim resection and patellar cartilage reconstruction (Table 2). On the other hand, it is the first arthroscopic technique to address isolated PC in patients without the clinical signs of patellofemoral instability. Thus, it carries all the benefits of an arthroscopic procedure, such as faster recovery, reduced pain and risk of complications, and higher precision owing to image magnification. It is so far the only procedure that addresses patellar cartilage lesions and the supposed biomechanical reason that led to their creation (supratrochlear rim). It is also the only alternative to the clinical state of the art of patellar cartilage reconstruction, surgical lowering of the patella, and distalizing tibial tubercle osteotomy, which are performed in this group of patients with mixed clinical outcomes.

**Table 2:** Advantages and Disadvantages of Presented Technique

Advantages
Minimally invasive procedure without need for wide arthrotomy; includes all benefits of arthroscopic procedures (reduced pain, faster recovery, reduced risk of complications, higher precision owing to image magnification)
Only alternative to patellar cartilage reconstruction, surgical lowering of patella, and distalizing tibial tubercle osteotomy
Use of 4 surgical ports allows accurate view of knee joint and access to supratrochlear rim
Disadvantages
Technically difficult procedure, requiring high arthroscopic skills
Requires specific dedicated tools for defect preparation, cartilage reconstruction, and rim resection
AMIC+ method requires use of dry arthroscopy, which may result in limited visibility during procedure

AMIC+, autologous matrix-induced chondrogenesis using bone marrow aspirate.

Distalizing tibial tubercle osteotomy has been reported as an effective treatment for patients with symptomatic patella alta and no severe patellofemoral dysplasia.^30^, ^31^, ^32^ This osteotomy aims to increase the contact area between the articular surface of the patella and femur, and hence the stability of the patellofemoral joint. However, this may lead to greater pressure on the patella, which is associated with further damage to the cartilage of the patellofemoral joint.^33^ It is possible that in the future, both techniques combined could lead to positive clinical outcomes in isolated PC treatment.

## Disclosures

All authors (T.P., B.B., J.B., A.B.) declare that they have no known competing financial interests or personal relationships that could have appeared to influence the work reported in this paper.

## Supplementary Data

Video 1 Surgical technique of rim resection. A supratrochlear rim and patellar defect are identified on magnetic resonance imaging (MRI) slices in the sagittal view of the patient’s left knee. The supratrochlear rim is delineated and its height is measured on the sagittal slice. A line is drawn parallel to and in line with the frontal femoral cortex. The height of the supratrochlear rim is the distance between the most anterior part of the supratrochlear rim and the line parallel to the frontal femoral cortex. The rim height should not exceed 3 mm. The patient is lying supine with an Esmarch tourniquet, and the surgical field and drapes are prepared in a sterile manner. The anterolateral (AL) portal is created, followed by the anteromedial (AM) portal, which is opened under direct arthroscopic visualization. Then, 2 additional portals are placed: the first suprapatellar portal, on the lateral side, and the second suprapatellar portal, on the medial side. First, diagnostic arthroscopy is performed with an examination of all knee compartments. Concomitant injuries and pathologies are addressed initially. Then, with the knee fully extended, the patellar defect is debrided with a shaver and Chondrectom instruments to the subchondral bone. The extent of the patellar defect is established. For this step of the procedure, the AL portal is used for viewing and the AM portal is used as a working portal. Then, the supratrochlear rim is addressed. The supratrochlear lateral portal is used for viewing, and the supratrochlear medial portal is used as a working portal. The cartilage covering the supratrochlear rim is resected with a shaver and arthroscopic ablation until the subchondral bone is fully exposed and the bony supratrochlear rim is fully visible. A dynamic test investigates the correlation between the patellar defect and the supratrochlear rim under direct arthroscopic visualization. The knee is flexed to approximately 30°, and the proximity between both of the aforementioned defects is viewed. The supratrochlear rim is resected using a straight burr (5.5-mm Dyonics Elite Abrader). The surface of the upper medial edge of the trochlear groove is smoothed, using the frontal femoral cortex as a reference point for alignment. Meanwhile, the Chondro-Gide collagen membrane is cut with a round cutter. A bone marrow needle is introduced through the AM portal into the intercondylar notch and bone marrow is aspirated. Prepared Chondro-Gide circles are soaked in bone marrow aspirate and introduced via dry arthroscopy into the previously prepared patellar defect and then onto the femoral surface in place of the resected rim. Finally, fibrin glue is used to stabilize the membrane on both sides. All adhesions of the glue with surrounding soft tissues must be resected with scissors; no adherence is allowed. After the glue dries, 10 cycles of passive flexion and extension are performed. The arthroscope is introduced for the final assessment of the membrane. The wounds are stitched, and a layered dressing is applied.
